# Identifying the Association Rules between Clinicopathologic Factors and Higher Survival Performance in Operation-Centric Oral Cancer Patients Using the Apriori Algorithm

**DOI:** 10.1155/2013/359634

**Published:** 2013-07-25

**Authors:** Jen-Yang Tang, Li-Yeh Chuang, Edward Hsi, Yu-Da Lin, Cheng-Hong Yang, Hsueh-Wei Chang

**Affiliations:** ^1^Department of Radiation Oncology, Faculty of Medicine, College of Medicine, Kaohsiung Medical University, Kaohsiung, Taiwan; ^2^Department of Radiation Oncology, Kaohsiung Medical University Hospital, Kaohsiung, Taiwan; ^3^Cancer Center, Kaohsiung Medical University Hospital, Kaohsiung Medical University, Kaohsiung, Taiwan; ^4^Department of Chemical Engineering and Institute of Biotechnology and Chemical Engineering, I-Shou University, Kaohsiung, Taiwan; ^5^Department of Medical Research, Kaohsiung Medical University Hospital, Kaohsiung, Taiwan; ^6^Department of Electronic Engineering, National Kaohsiung University of Applied Sciences, Kaohsiung, Taiwan; ^7^Department of Biomedical Science and Environmental Biology, Kaohsiung Medical University, Kaohsiung, Taiwan

## Abstract

This study computationally determines the contribution of clinicopathologic factors correlated with 5-year survival in oral squamous cell carcinoma (OSCC) patients primarily treated by surgical operation (OP) followed by other treatments. From 2004 to 2010, the program enrolled 493 OSCC patients at the Kaohsiung Medical Hospital University. The clinicopathologic records were retrospectively reviewed and compared for survival analysis. The Apriori algorithm was applied to mine the association rules between these factors and improved survival. Univariate analysis of demographic data showed that grade/differentiation, clinical tumor size, pathology tumor size, and OP grouping were associated with survival longer than 36 months. Using the Apriori algorithm, multivariate correlation analysis identified the factors that coexistently provide good survival rates with higher lift values, such as grade/differentiation = 2, clinical stage group = early, primary site = tongue, and group = OP. Without the OP, the lift values are lower. In conclusion, this hospital-based analysis suggests that early OP and other treatments starting from OP are the key to improving the survival of OSCC patients, especially for early stage tongue cancer with moderate differentiation, having a better survival (>36 months) with varied OP approaches.

## 1. Introduction

 In Taiwan, betel nut chewing, cigarette smoking, and alcohol consumption have been found to be highly associated with oral cancer [1], with habitual betel nut chewers showing a particular high prevalence [2–4]. Oral cancer is one of the 10 most prevalent cancers in Taiwan, mostly classified as oral squamous cell carcinoma (OSCC) [5], which has high rates of morbidity and mortality [6] because diagnosis often only takes place in the later stages [7]. Although many tumor markers [8–10] and single nucleotide polymorphism (SNP) markers [11] have been reported as being associated with oral cancer, outcome-based studies focusing on oral cancer therapy are lacking.

The survival of OSCC patients following surgical therapy has been reported to be affected by tumor size, nodal metastasis, staging, and differentiation [12]. Some researchers have been further concerned with factors involved in outcomes for postoperative radiotherapy for OSCC patients [13]. However, the correlation between the multiple survival affecting factors for predicting the well survival of OSCC therapy is less addressed and remains a challenge.

Recently, several computational methodologies have been introduced to analyze the relationship between multiple factors and therapies for several non-OSCC diseases, including machine learning algorithms [14], data mining [15], decision tree-based learning [16], and rule-based multiscale simulations [17]. 

The Apriori algorithm is used here to explore the correlation between clinical factors and good survival outcomes (i.e., >36 months) in operation- (surgery-) centric treatments, including operation alone, operation/IA, and operation/IA, CT, IV, and RT, where IA, IV, CT, and RT, respectively stand for intra-arterial, intravenous, oral chemotherapies, and radiotherapy. The study aims to computationally evaluate the correlation between clinicopathological factors and survival outcomes in 493 OSCC patients treated by operation alone or by operation followed with other nonsurgical treatments.

## 2. Materials and Methods

### 2.1. Data Source

The database used to construct our cases and control groups was obtained from the chart registry of cancer center of the Kaohsiung Medical University Hospital from 2004 to 2010. Patients were excluded if they had distant metastases at presentation, did not complete the therapeutic protocol in Kaohsiung Medical University Hospital, or had incomplete records. A total of 493 patients fulfilled the requirements and were included for further analyses (the raw data set is available at http://bioinfo.kmu.edu.tw/OP_high-OP_low_groups.xlsx). The patients were followed at Kaohsiung Medical University Hospital. The last followup was recorded from the last outpatient visit or the date of death. This use of patient data and the study design were reviewed and approved by the Institutional Review Board of Kaohsiung Medical University Hospital (KMUH-IRB-EXEMPT-20130029).

### 2.2. Introduction of the Apriori Algorithm

The problem for association rule learning can be stated as follows. Let *I* = {*i*_1_, *i*_2_,…, *i*_*m*_} be a set of literals, called items. Let transaction *T* be a set of items, where *T*⊆*I*. Let *D* be a set of transactions. The objective of the association rule is an implication of the form *A*⇒*B*, where *A* ⊂ *I* and *B* ⊂ *I*, if *A*∩*B* = *Ø*. The rule *A*⇒*B* holds in the transaction set *D* with *confidence c* if *c*% of transactions in *D* that contain *A* also contain *B*. The rule *A*⇒*B* has support *s* in the transaction set *D* if *s*% of transactions in *D* contain *A* ∪ *B*. Item sets with the minimum support *s* are called large itemsets, and the others small itemsets.

 The Apriori algorithm was proposed by Agrawal and Srikant in 1994 [18] and has been widely used for frequent itemset mining and association rule learning in databases. The Apriori algorithm aims to generate the desired rules from large itemsets. The general idea is that if items *ABCD* are large itemsets, then any rule in *ABCD* will have the minimum required support because *ABCD* is large; that is, *AB*⇒*CD*.

 The Apriori algorithm can be divided into three steps.  Algorithm 1 shows the pseudocode of the Apriori algorithm. The algorithm's first pass counts item occurrences to screen the large itemsets (Section 2.2.1). The second pass generates the candidate itemsets *C*_*k*_ from large itemsets *L*_*k*−1_, using the apriori-gen function (Section 2.2.2). Next, each transaction *t* checks whether the subsets of *k*-itemsets of *t* belong to *C*_*k*_, called subset function and described in Section 2.2.3. Finally, each *c* counts item occurrences in *C*_*t*_, and *c* will be stored in *L*_*k*_ if *c.count* minimum support. The algorithm terminates when *L*_*k*_ is empty; that is, no frequent set of *k* or more items is present in *D*. 

#### 2.2.1. Screening the Large 1-Itemsets

Algorithm 2 shows the pseudo code of first pass which simply counts item occurrences *I* = {*i*_1_, *i*_2_,…, *i*_*m*_} to determine the large itemsets in all items. The array of *item counts* is used to count item occurrences, and elements in *Item-counts* having minimum support are included in the *L*_1_ set. 

#### 2.2.2. Candidate Set Generations

The function apriori-gen (*L*_*k*−1_) generates *C*_*k*_ from *L*_*k*−1_, and it returns a superset of the set of all large *k*-itemsets.  Algorithm 3 shows the pseudo code of the function apriori-gen (*L*_*k*−1_). We use a set *c*, *c* = {*L*_*k*−1_.*item*[*i*]}, for all *i* ∈ {1,…, *k* − 1}, to store the frequent (*k* − 1)-itemsets in *L*_*k*−1_. The selections of the pairs are called *L*_*k*−1_.*item*_*p*_, *L*_*k*−1_.*item*_*q*_ ∈ *L*_*k*−1_. For each *L*_*k*−1_.*item*_*p*_ in *L*_*k*−1_, we start the search tuples in the *L*_*k*−1_.*item*_*p*_ and stop the search if we find *L*_*k*−1_.*item*_*q*_ such that 1 to *k* − 2 items are not equal to the 1 to *k* − 2 items of *L*_*k*−1_.*item*_*p*_. Only if we find an *L*_*k*−1_.*item*_*q*_ that satisfies *L*_*k*−1_.*item*_*p*_[*i*] = *L*_*k*−1_.*item*_*q*_[*i*], for all *i* ∈ {1,…, *k* − 2}, the *c* does create the *k*-itemset = {*L*_*k*−1_.*item*_*p*_[*i*],…, *L*_*k*−1_.*item*_*p*_[*k* − 2], *L*_*k*−1_.*item*_*p*_[*k* − 1], *L*_*k*−1_.*item*_*q*_[*k* − 1]}. Finally, *c* checks whether the subsets of *c* are included in *L*_*k*−1_.

#### 2.2.3. Candidate Set Counts Using Hash Tree

After the candidate sets *C*_*k*_ are generated, the *C*_*k*_ are stored in a hash tree created by the function subset (*C*_*k*_, *t*). The leaf of the hash tree comprises the pointers to *C*_*k*_ and the associated counters, and the leaf refers to distinct partitions of *C*_*k*_. In the hash tree, the hash function can be used to insert the candidate itemsets and search the transaction subsets in *C*_*k*_. The hash function is hash(*i*) = *i*mod⁡*T*, *T* < *m*, where *T* is a constant, and *m* is the number of items. Function subset (*C*_*k*_, *t*) is a recursive function which traverses the tree starting from the root node to the leaves, with each item in *t* = {*i*_1_,…, *i*_*d*_} chosen as a possible starting item of a candidate itemset. It is applied at every level of the tree. When *t* reaches a leaf of the tree, all candidate itemsets are checked against *t* and their counters are updated.

### 2.3. Statistics Analysis

Statistical analysis was performed with JMP version 9. All statistical tests were done at a 0.05 significance level.

## 3. Results and Discussion

### 3.1. Demographic Data and Survival

#### 3.1.1. Age and Survival

As shown in Table 1, all patients were categorized into 2 groups based on whether the survival is greater or less than 36 months. In this regard, no difference in varied age groups can be found. This is probably because anyone who was eligible for surgical resection would have comparable survival rates.

#### 3.1.2. Subsites and Survival

As shown in Table 1, the site distribution of the 493 cases of oral cancer patients showed common affected sites including the cheek mucosa, gum, tongue, and retromolar trigon. Postsurgical organ function and cosmetics may vary with surgical site, but no difference to survival could be found.

#### 3.1.3. Laterality and Survival

As shown in Table 1, laterality is recorded in the database of cancer registries and is a mixed expression of clinical/pathological tumor size and location. It does not play a significant role in the surgical group.

#### 3.1.4. Grade and Survival

As shown in Table 1, comparison of the pathological characteristics between >5-year (*n* = 271) and <5-year survival (*n* = 222) revealed better treatment outcomes for low grade tumors (*P* = 0.0006), suggesting that well-differentiated tumors are less aggressive and thus are associated with better overall survival.

#### 3.1.5. Regional Lymph Nodes and Survival

As shown in Table 1, regional lymph node examination might express the details and quality of surgical resection. However, the number of examined lymph nodes was not found to have an effect on survival. This might be due to cross-interaction between clinical lymph node stages and overall survival.

#### 3.1.6. Clinical Stages, Pathology Stages, Clinical/Pathology Tumor Sizes, and Survival

As shown in Table 1, neither clinical nor pathological stages were found to have an impact on 5-year survival. There might be some influencing factors between low- and high-tumor stages which cannot be simply explained by surgery. However, for clinical/pathological tumor size alone, significant differences between >5-year and <5-year groups are found (*P* = 0.0004 and *P* = 0.0141, resp.). Smaller tumor size means less tumor burden and has less surrounding tissue infiltration, which may explain improved overall outcomes.

#### 3.1.7. Surgical Modalities and Survival

As shown in Table 1, treatment modalities (OP) were further differentiated into 3 groups based on different adjuvant therapies, that is, surgery alone, surgery plus intra-arterial chemotherapy, and surgery plus concomitant chemoradiotherapy. Significant differences between groups were found (*P* < 0.0001), and further analysis of surgical modalities based on the clinical/pathological stages could produce interesting insights.

This hospital-based study followed nearly 500 patients with oral squamous cell carcinoma after surgical treatment. Results showed that age of onset and laterality of tumor location did not influence the treatment outcome. The latter might be attributed to oral cancer being a less multifocal or multicentric disease than, for example, breast cancer and, hence, laterality of the primary tumor has less influence on survival. These findings are in line with previous findings [19, 20].

Advanced tumor stage or failure of locoregional control negatively influences survival in patients with OSCC [21]. However, we did not observe a significant influence from either clinical or pathological tumor stages. Similar to our findings, Pandey et al. reported no difference in survival rates for the extent of tumor [22], and the observed difference might be due to the facts that all stages of tumor have been poured in the analysis.

In the present study, multimodality treatment proved to be a prognostic factor. Benefit from systemic or adjuvant local therapies might correlate with disease biology as the grade of tumor differentiation was also an important influencing factor.

### 3.2. Data Mining Results Using Apriori Algorithm

Table 2 shows the best rules for OP > 36 months. The head *Y* and body *X* represent a class association rule *X*⇒*Y* which means the head *Y* of an association rule *X*⇒*Y* (with rule body *X*) must be restricted to one attribute-value pair. The attribute of the attribute-value pair is thus the class attribute. The resulting rules can be evaluated according to three metrics: confidence, lift, and leverage. The minimum value of 1.5 for lift (or improvement) is computed as the confidence of the rule divided by the support of the right-hand-side (RHS). The lift represents the ratio of probability. Given a rule *X*⇒*Y*, *X* and *Y* occur together to the multiple of the two individual probabilities for *X* and *Y*; that is,

(1) $$\begin{array}{c} \text{lift}=\frac{\text{Pr}\left(X,Y\right)}{\text{Pr}\left(L\right)\cdot \text{Pr}\left(Y\right)}. \end{array}$$

If lift is 1, *X* and *Y* are independent. The higher lift is above 1, the more likely that the existence of *X* and *Y* together in a transaction is due to a relationship between them and not just random occurrence. Unlike lift, leverage measures the difference between the probability of co-occurrence of *X* and *Y* as the independent probabilities of each of *X* and *Y*; that is,

(2) $$\begin{array}{c} \text{leverage}=\text{Pr}\left(X,Y\right)-\text{Pr}\left(X\right)\cdot \text{Pr}\left(Y\right). \end{array}$$

Leverage measures the proportion of additional cases covered by both *X* and *Y* above those expected if *X* and *Y* were independent of each other. Thus, for leverage, values above 0 are desirable whereas values greater than 1 are desirable for lift. Finally, conviction is similar to lift, but it measures the effect of the right-hand side not being true and also inverts the ratio. Conviction is measured as

(3) $$\begin{array}{c} \text{conviction}=\frac{\text{Pr}\left(X\right)\cdot \text{Pr}\left(\text{not}\;Y\right)}{\text{Pr}\left(X,Y\right)}. \end{array}$$

Table 2 shows that the rule “grade/differentiation = 2 and clinical stage group = early” is associated with the rule “primary site = tongue and group = OP.” The rule shows 49 patients as being grade/differentiation = 2 and clinical stage group = early, while 27 of these 49 patients fulfill the rules “primary site = tongue and group = OP.” The confidence shows the proportion of the rule “primary site = tongue and group = OP” in the rule “grade/differentiation = 2 and clinical stage group = early,” that is, 27/49. The lift is 1.91, meaning the existence of rule “grade/differentiation = 2 and clinical stage group = early” and rule “primary site = tongue and group = OP” together in a transaction is not just a random occurrence. The leverage value of 0.05 means that the proportion of additional cases covered by both rule “grade/differentiation = 2 and clinical stage group = early” and rule “primary site = tongue and group = OP” are greater than those that would be expected if these two rules were independent of each other. The conviction value of 1.52 indicates the effect of the right-hand side is not being true.

From the top down in Table 2, the lift values gradually decrease but still show a high correlation between the body/head and survival of >36 months. When the Apriori algorithm-based lift value of the items listed in “body” and “head” of Table 2 is high, there is less chance of misinterpretation of the relationships between each item. Judging by the top 8 results, the same items such as grade/differentiation = 2, clinical stage group = early, primary site = tongue, and group = OP flowed between the “body” and “head”. These data suggest that early stage tongue cancer with moderate differentiation will have a better survival (>36 months) with varied surgical approaches where the OP has three kinds of treatments. 

Judging by the top 9 to 10 results, however, only three items are included without the group = OP and their lift values are decreased to 1.74. These results suggest that the factor of “group = OP” is not important to the top 9 to 10 results and is less strongly correlated compared with the top 8 results. It also implies that the OP plays an important role in creating a correlation with improved survival (>36 months). In clinical settings, this might be due to good treatment outcome which often accompanies surgery.

Accordingly, our proposed Apriori algorithm is a relatively simple form of rule-based computation to identify potential rules involving various factors, such as grade/differentiation = 2, clinical stage group = early, primary site = tongue, and group = OP. The algorithm can reveal the combination effect of these factors on the outcome of OSCC therapy.

## 4. Conclusion

This hospital-based analysis reviewed 493 patients with OSCC to mine survival factors in operation-centric patients. The results identify the importance of grade/differentiation = 2, clinical stage group = early, primary site = tongue, and group = OP in predicting higher survival for OSCC patients.

## Figures and Tables

**Algorithm 1:**
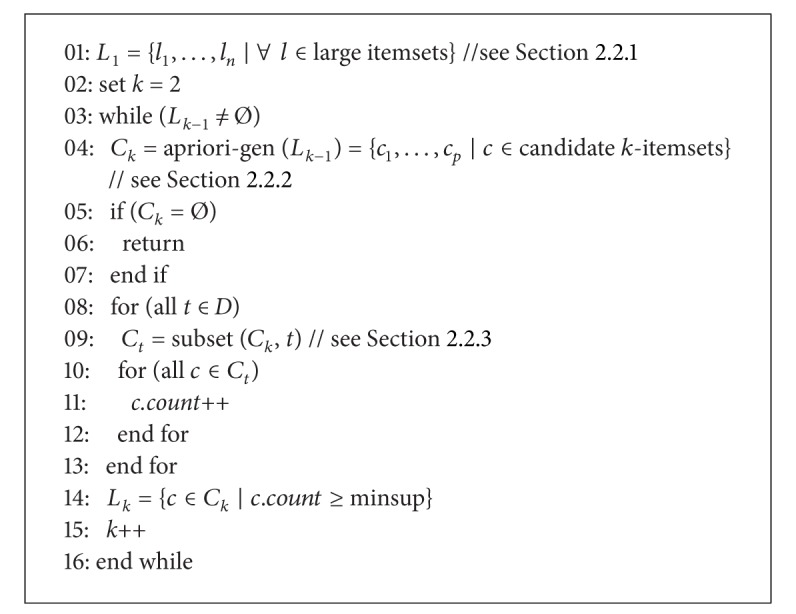
Pseudocode of the Apriori algorithm.

**Algorithm 2:**
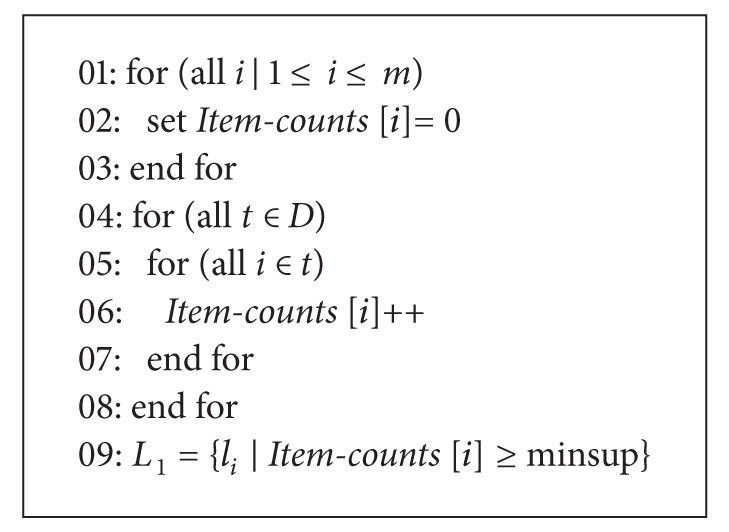
The first pass of the Apriori algorithm.

**Algorithm 3:**
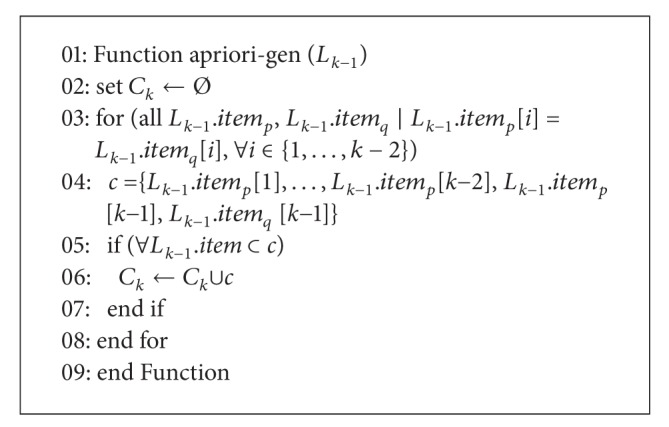
Pseudocode of the function apriori-gen().

**Table 1:** Demographic data of 493 enrolled patients with OSCC.

Characteristics	Survived months			*P *value^∗1^	5-year survival (%)	*P *value^∗2^
	Total	>36 group	<36 group			
Age				0.7786		0.5556
<30	7	3	4		71.4	
30~50	228	125	103		77.2	
50~70	236	129	107		79.2	
>70	22	14	8		63.6	
Primary Site				0.7915		0.1957
Lip	36	24	12		86.1	
Cheek mucosa	184	103	81		83.2	
Gum	42	25	17		71.4	
Tongue	175	88	87		72.0	
Mouth floor	19	11	8		68.4	
Palate	5	3	2		60.0	
Retromolar	27	15	12		77.8	
Vestibule	2	1	1		100.0	
Nonspecific	3	1	2		100.0	
Laterality^∗3^				0.3965		0.8612
00	37	22	15		73.0	
01	230	123	107		79.1	
02	223	123	100		76.7	
03	3	3	0		66.7	
04	0	0	0		NA	
Grade/differentiation				0.1476		**0.0006**
01	287	156	131		80.1	
02	123	60	63		65.0	
03	7	5	2		57.1	
04	1	1	0		100.0	
09	75	49	26		89.3	
Regional lymph nodes examined				0.1550		0.1424
<5	285	160	125		80.4	
>10	134	65	69		73.1	
5~10	73	45	28		74.0	
Clinical stage group				0.0749		0.5689
Stage 0	4	0	4		75.0	
Stage 1	141	79	62		80.1	
Stage 2	73	47	26		71.2	
Stage 3	131	69	62		77.1	
Stage 4	82	50	32		72.0	
Pathologic stage group				0.2540		0.0514
Stage 0	2	2	0		100.0	
Stage 1	215	112	103		82.3	
Stage 2	92	52	40		75.0	
Stage 3	31	15	16		74.2	
Stage 4	58	24	34		67.2	
Clinical tumor size				0.3967		**0.0004**
<2 cm	162	100	62		87.0	
2~4 cm	244	134	110		71.3	
>4 cm	33	19	14		66.7	
Pathology tumor size				0.4417		**0.0141**
<2 cm	197	114	83		81.7	
2~4 cm	183	94	89		69.4	
>4 cm	25	14	11		72.0	
OP group^∗4^				**<0.0001**		**<0.0001**
01	385	238	147		81.6	
02	27	14	13		66.7	
03	81	19	62		61.7	

^∗1^ *P* value for the comparison of the survival between >36 and <36 months groups.

^∗2^ *P* value for 5-year survival among the items of the same characteristics group.

^∗3^0: unknown primary site or the shape of the organ is not paired; 1: the primary site is originated from the right side; 2: the primary site is originated from the left side; 3: only one side is invaded but it is not clear which side (R't or L't) it is originated from; 4: both sides are invaded but the origin of the primary site is not clear and the chart record describes only one primary site.

^∗4^OP group for 01: OP only; 02: OP→IA; 03: OP→CT, OP→CT + IV, OP→CT→RT, OP→IA→RT, OP→IV,OP→IV→RT, OP→RT, OP→RT + CT,OP→RT + IV, OP→RT→CT, OP→RT→IA, OP→RT→IV. Symbols: OP: operation; IA: intraarterial chemotherapy; CT: oral chemotherapy; IV: intravenous chemotherapy; RT: radiotherapy; →: then.

**Table 2:** Ranking of the top 10 best rules found in survival larger than 36 months.

Body^∗1^	No.	Head^∗1^	No.	Confidence	Lift^∗2^	Leverage	Conviction
Grade/differentiation = 2 Clinical stage group = early	49	Primary site = tongue Group = OP	27	0.55	1.91	0.05	1.52
Primary site = tongue Group = OP	78	Grade/differentiation = 2 Clinical stage group = early	27	0.35	1.91	0.05	1.23
Primary site = tongue Clinical stage group = early	70	Grade/differentiation = 2 Group = OP	27	0.39	1.9	0.05	1.27
Grade/differentiation = 2 Group = OP	55	Primary site = tongue Clinical stage group = early	27	0.49	1.9	0.05	1.41
Grade/differentiation = 2	60	Primary site = tongue Clinical stage group = early Group = OP	27	0.45	1.88	0.05	1.34
Primary site = tongue Clinical stage group = early Group = OP	65	Grade/differentiation = 2	27	0.42	1.88	0.05	1.3
Primary site = tongue	88	Grade/differentiation = 2 Clinical stage group = early Group = OP	27	0.31	1.81	0.04	1.18
Grade/differentiation = 2 Clinical stage group = early Group = OP	46	Primary Site = tongue	27	0.59	1.81	0.04	1.55
Grade/differentiation = 2	60	Primary site = tongue Clinical stage group = early	27	0.45	1.74	0.04	1.31
Primary site = tongue Clinical stage group = early	70	Grade/differentiation = 2	27	0.39	1.74	0.04	1.24

^∗1^Stages 0 to 3 of clinical stage group and pathologic stage group as shown in Table 1 are regarded as early and stage 4 is regarded as late stage in Table 2.

^∗2^The best rules with lift >1.5 were shown here.
